# White matter deficits in cocaine use disorder: convergent evidence from in vivo diffusion tensor imaging and ex vivo proteomic analysis

**DOI:** 10.1038/s41398-021-01367-x

**Published:** 2021-04-29

**Authors:** Lucca Pizzato Tondo, Thiago Wendt Viola, Gabriel R. Fries, Bruno Kluwe-Schiavon, Leonardo Mello Rothmann, Renata Cupertino, Pedro Ferreira, Alexandre Rosa Franco, Scott D. Lane, Laura Stertz, Zhongming Zhao, Ruifeng Hu, Thomas Meyer, Joy M. Schmitz, Consuelo Walss-Bass, Rodrigo Grassi-Oliveira

**Affiliations:** 1 Developmental Cognitive Neuroscience Lab (DCNL), Brain Institute, Pontifical Catholic University of Rio Grande do Sul (PUCRS), Porto Alegre, Brazil; 2 Louis A. Faillace, MD, Department of Psychiatry and Behavioral Sciences, University of Texas Health Science Center at Houston, Houston, TX USA; 3 Department of Psychiatry, University of Vermont, Burlington, VT USA; 4 Nathan Kline Institute and Child Mind Institute, New York, NY USA; 5 Center for Precision Health, School of Biomedical Informatics, University of Texas Health Science Center at Houston, Houston, TX USA; 6 Translational Neuropsychiatry Unit, Department of Clinical Medicine, Aarhus University, Aarhus, Denmark

**Keywords:** Biomarkers, Neuroscience

## Abstract

White matter (WM) abnormalities in patients with cocaine use disorder (CUD) have been studied; however, the reported effects on the human brain are heterogenous and most results have been obtained from male participants. In addition, biological data supporting the imaging findings and revealing possible mechanisms underlying the neurotoxic effects of chronic cocaine use (CU) on WM are largely restricted to animal studies. To evaluate the neurotoxic effects of CU in the WM, we performed an in vivo diffusion tensor imaging assessment of male and female cocaine users (*n* = 75) and healthy controls (HC) (*n* = 58). Moreover, we performed an ex vivo large-scale proteomic analysis using liquid chromatography-tandem mass spectrometry in postmortem brains of patients with CUD (*n* = 8) and HC (*n* = 12). Compared with the HC, the CUD group showed significant reductions in global fractional anisotropy (FA) (*p* < 0.001), and an increase in global mean (MD) and radial diffusion (RD) (both *p* < 0.001). The results revealed that FA, RD, and MD alterations in the CUD group were widespread along the major WM tracts, after analysis using the tract-based special statistics approach. Global FA was negatively associated with years of CU (*p* = 0.0421) and female sex (*p* < 0.001), but not with years of alcohol or nicotine use. Concerning the fibers connecting the left to the right prefrontal cortex, Brodmann area 9 (BA9), the CUD group presented lower FA (*p* = 0.006) and higher RD (*p* < 0.001) values compared with the HC group. A negative association between the duration of CU in life and FA values in this tract was also observed (*p* = 0.019). Proteomics analyses in BA9 found 11 proteins differentially expressed between cocaine users and controls. Among these, were proteins related to myelination and neuroinflammation. In summary, we demonstrate convergent evidence from in vivo diffusion tensor imaging and ex vivo proteomics analysis of WM disruption in CUD.

## Introduction

Cocaine use disorder (CUD) is a chronic disease characterized by compulsive drug use with negative psychosocial consequences, affecting ~0.5–3% of the adult population worldwide^1,2^. It is a severe, complex, and debilitating illness leading to higher mortality rates and several daily life disturbances^3–5^. In the brain, CUD is associated with decreased dopamine signaling and widespread structural and functional alterations^6^.

Although most neuroimaging studies have examined the chronic cocaine use (CU) effects on specific gray matter brain regions, several recent investigations have focused on revealing the white matter (WM) signature of CUD^7^. Diffusion-weighted imaging (DWI) is a magnetic resonance sequence that captures water diffusion properties in the brain and has been frequently used in studies that determined the fibers and tracts that are progressively deteriorated because of repeated cocaine consumption. In addition, diffusion tensor imaging (DTI) is a widely used method to model the DWI signal^8^, which allows the in vivo assessment of WM microstructural characteristics through the analysis of eigenvalues and eigenvectors. These eigenvectors are used to derive the four main metrics analyzed in DTI studies: fractional anisotropy (FA), axial diffusion (AD), radial diffusion (RD), and mean diffusion (MD). Generally, properly myelinated fibers present high FA and low RD and MD values^9^. FA changes can reflect the coherence of fibers, microglia, axon packing, density, myelination, or inflammation^10^, and its decreased signal has been associated with many diseases affecting the WM integrity^11^.

Previous studies have supported that CUD is associated with decreased FA signal^12–15^. A recent meta-analysis of data obtained from DTI studies that included patients with stimulant use disorder revealed a small-to-moderate group-effect in reducing the FA values; conversely, a sub-analysis focusing on patients with CUD showed greater heterogeneity of findings and effect sizes^16^. Another meta-analysis including only data from tract-based special statistics (TBSS) studies for CUD revealed a decreased FA in the genu of the corpus callosum and a tenuous effect of pre-imaging abstinence on FA values^17^. Regarding the clinical associations, a study has found that the decrease in FA values is correlated with the years of CU^18^. Other studies have shown a positive association between the FA values at treatment onset and the abstinence duration during cocaine addiction treatment^15,19^. Moreover, cognitive impairments in decision-making^20^ and working memory^21^ have been associated with altered DTI metrics among cocaine users.

However, the heterogeneous distribution of DTI findings in patients with CUD highlights the challenges and limitations of the currently available research. First, most studies have been conducted including mainly male participants, in which the CUD group is generally composed of <40 individuals^16^. This may result in difficulties in obtaining precise DTI comparisons between users and non-users beyond the effect sizes of major callosal tracts and limited evidence regarding the female cocaine users. In addition, although some studies have documented clinical associations with DTI metrics, small sample sizes generate additional obstacles for such analyses, especially when accounting for covariate effects (e.g., age and sex). Second, >60% of individuals with CUD report chronic alcohol use consumption^22–25^. The high comorbidity profile suggests that the evaluation of samples obtained from polysubstance users is important, especially for the generalization of the findings to the general population^26^. Third, biological investigations that support the imaging findings and reveal possible mechanisms underlying the chronic CU neurotoxic effects on the WM are largely restricted to animal studies. Some of these studies have suggested that the reduced myelin-related protein levels in the brain may correlate with the decreased FA signal in the corpus callosum^27,28^, despite human studies using postmortem brain tissues have identified several neurobiological changes in chronic cocaine users^29^, particularly decreased myelin-related gene expression, suggesting an altered myelin structure and integrity^30^. DWI measures rely on multiple factors such as axonal coherence, membrane permeability, and myelination^10^, and their alteration cannot be interpreted, by itself, as conclusive evidence of WM impairment: proteomic and histological findings corroborating neuroimage data are essential.

Considering these limitations, we performed a comprehensive study combining two distinct approaches that evaluated the CUD neurotoxic effects in brain WM. First, we compared the DTI metrics between individuals with and without CUD, including male and female individuals, using two complementary post-processing techniques: TBSS and deterministic tractography^31^. Moreover, we recruited a larger number of participants than usual for DTI studies. First, we focused on crack-cocaine-dependent individuals, most of whom report concurrent tobacco and alcohol use, to meaningfully capture a more representative sample of polysubstance users^32,33^. Then, we assessed the protein expression levels in the brain of individuals with and without CUD to determine whether alterations in pathways related to WM integrity in the human brain are affected by chronic CU. To that end, we performed a large-scale proteomic analysis in postmortem brains. We hypothesized that an investigation combining in vivo imaging and ex vivo biochemical approaches would extend previous findings and better explain the biological mechanisms associated with WM morphological deficits in patients with CUD.

## Methods

The in vivo imaging examinations were performed in patients with CUD during the acute abstinence phase of smoked cocaine (crack-cocaine) detoxification treatment. The ex vivo postmortem brain study was performed using brain tissues collected from individuals whose families donated their brains to the UTHealth Brain Collection for Research in Psychiatric Disorders (Houston, TX, USA). The ethics committees of the institutions involved (PUCRS and UTHealth) in both studies approved the research protocol. This study was conducted in accordance with the Declaration of Helsinki. All participants (in vivo study) or their families (ex vivo study) provided written informed consent after receiving a complete description of the study.

### In vivo neuroimaging sample

The participants were 75 patients with CUD who were recruited during inpatient detoxification and drug rehabilitation treatment in southern Brazil. The treatment consisted of psychoeducation and support groups, moderate physical activity, a balanced diet (2200 Kcal/day), nursing care, and psychological and medical treatments. The patients remained in a controlled environment without access to alcohol, illicit drugs, and tobacco. Prior to neuroimaging assessment, we collected urine samples from the participants to test for cocaine, benzodiazepines, cannabis, amphetamine, and opiates using an instant urine drug test (EDOAP754, Easy Healthcare Corporation, Burr Ridge, IL, USA). The inclusion criteria for the cocaine group were as follows: (1) diagnosis of CUD according to the Structured Clinical Interview for DSM-IV Axis I Disorders (SCID-IV)^34^; (2) IQ > 80 based on the Wechsler Abbreviated Scale of Intelligence-II^35^; (3) age, 18–45 years; (4) right-handed; (5) a positive urine test for cocaine at admission; and (6) reporting cocaine as the preferred drug.

All participants had all medications suspended and tested negative for cocaine, cannabis, amphetamines, opioids, and benzodiazepines on the day of the magnetic resonance imaging (MRI) examination, which occurred 2 weeks after admission to the detoxification unit.

Fifty-eight healthy and unmedicated control participants (healthy controls; HC) with a similar age and socio-economic background were included by convenience sampling (advertising). Eligible participants were those with: (1) a negative history of use of any illicit drug; (2) no psychiatric diagnosis except for nicotine use according to the SCID-IV axis I.

The exclusion criteria were as follows: (1) positive drug urine test on the day of MRI examination; (2) restrictions related to MRI procedures; (3) current psychotic symptoms; (4) neurologic disorders; and (5) presence of human immunodeficiency virus or syphilis.

The demographic characteristics of the participants are presented in Table 1.

**Table 1:** Sociodemographic and clinical characteristics of the samples.

	Neuroimaging sample			
	CUD (*n* = 75) (mean/%, SD/*N*)	Controls (*n* = 58) (mean/%, SD/*N*)	Statistics (*df*)	*p*
*Sociodemographic*				
Age (years)	32.86 (7.08)	27.98 (7.12)	*t(131)* = −3.93	**>0.0001**
Sex (male)	49.3 (37)	36.2 (21)	*x*^*2*^*(1)* *=* 2.29	0.130
Ethnicity (whites)	44.6 (33)	65.5 (36)	*x*^*2*^*(3)* *=* 5.95	0.114
Education (years)	7.39 (2.75)	11.33 (1.23)	W = 3768	**>0.0001**
*ASI-6 (standardized T scores)*				
Drugs	52.08 (7.13)	-		
Family/child	55.96 (10.01)	-		
Alcohol	50.34 (8.85)	-		
Psychiatric status	50.50 (7.74)	-		
Medical	43.42 (8.11)	-		
Legal status	49.89 (5.71)	-		
Employment	38.94 (4.29)	-		
Family/social support	50.94 (12.15)	-		
Family/social problems	52.41 (8.96)	-		
*SCID-I (SUD–current)*				
Cannabis	29.3 (22)	-		
Snorted cocaine	14.7 (11)	-		
Inhalant	1.3 (1)	-		
Tobacco	73.3 (55)	5.1 (3)	*x*^*2*^*(1) =* 61.79	**>0.0001**
Alcohol	29.3 (22)	-		
*Years of regular drug use—mean (SD)*				
Tobacco	5.82 (9.10)	3.90 (8.8)	W = 1102	**>0.0001**
Alcohol	6.64 (7.66)	0.25 (0.94)	-	-
Cannabis	7.93 (7.63)	0.10 (0.45)	-	-
Cocaine	8.18 (6.38)	0 (0)	-	-
	Proteomics sample			
	CUD (*n* = 8) (mean/%, SD/*N*)	Controls (*n* = 12) (mean/%, SD/*N*)	Statistics (*df*)	*p*
*Sociodemographic*				
Age (years)	45.7 (9.72)	51.7 (13.81)	*W* = 61.50	0.082
Sex (male)	62.5 (5)	83.3 (10)	*x*^*2*^*(1) =* 1.11	0.292
Ethnicity (whites)	25 (2)	58.3 (7)	*Fisher exact*	0.197
Post-mortem intervals (hours)	24.1 (8)	29.4 (8.4)	*W* = 67	0.190

*SD* standard deviation, *t* student *t* test statistics; *χ*^2^ chi-squared, *W* Wilcoxon rank-sum test statistics.

Significant values in bold.

### Severity of drug use

To better characterize the CUD sample and to investigate the severity of drug use, the participants completed the Addiction Severity Index (ASI-6)^36^. The severity score for each domain is affected by the following conditions: drug use, family-related issues, alcohol consumption, psychiatric issues, medical problems, legal issues, financial problems, lack of social support, and social problems. They standardized that the T scores have a mean of 50 and a standard deviation of 10, theoretically ranging from 0 to 100. Higher scores indicate greater problem severity. The number of days of substance consumption prior to treatment enrollment (last 30 days) and the years of regular substance use (cannabis, tobacco, alcohol, and CU at least 3 days per week) were inquired^24^.

### Neuroimaging

See detailed information in Supplemental Methods.

T1-weighted anatomic and DWI images were acquired on a 3 T Signa GE scanner (GE Healthcare, Chicago, IL, USA) at the Brain Institute of Rio Grande do Sul, Brazil. FMRIB Software Library (FSL; Oxford, UK) version 6.0 was used to preprocess and extract FA, MD, AD, and RD maps from the DWI data set. Voxelwise statistical analysis of the FA data were performed using TBSS’s^37^ pipeline: brain masks were created with *bet*, eddy-current and head-movement distortions were corrected using affine registration of all gradient volumes with the *b* = 0 volume with *eddy*^37^, and estimation of DWI measures with *dtifit*. Between-group comparison and within crack-cocaine group analysis was performed FSL’s general linear model software *randomize*. The results were corrected with a threshold-free cluster enhancement approach (TFCE)^38^ and controlled for age, sex, and years of education. Whole-brain metrics were also analyzed. Considering TBSS’ methodological limitations, especially the intraparticipant variability for minor tracts and anatomical specificity of tracts within the skeleton^39^, we also conducted a tractography-based analysis with DSI Studio (http://dsi-studio.labsolver.org/) to investigate the connectivity of the cortical regions whose specific tracts passed through the affected WM regions in TBSS. The diffusion data for each participant were reconstructed in the corresponding T1 image and, therefore, the fiber tracking could be performed in the participants’ native space. DTI metrics for the tracts connecting Brodmann area 9 (BA9) with Brodmann area 8 (BA8) and connecting left and right BA9 were extracted and analyzed. Neuroimaging data have been deposited to the OpenNeuro repository (https://openneuro.org/datasets/ds003599/versions/1.0.0).

### Ex vivo postmortem brain sample

Brain tissue from 20 participants was collected postmortem from individuals diagnosed with CUD (*n* = 8) and from HC individuals without any known psychiatric disorders (*n* = 12). The samples were matched for age and postmortem interval (PMI). As part of the clinical information, the medical records were obtained and a detailed psychological autopsy was performed on all participants by interviewing the next-of-kin. Information regarding the psychiatric clinical phenotypes (evidence of depression, mania, and psychosis) stressful life events, age of drug use onset, types of drugs used, smoking and drinking history, and any co-morbidities, was obtained.

A CUD diagnosis was confirmed based on the psychological autopsy, detailed medical records, and review of all relevant case information by three psychiatrists at a consensus meeting. The cause of death was obtained from the medical examiner’s report and toxicological findings after death.

### Proteomics analysis

Frozen brain tissues (50 g) obtained from the dorsolateral prefrontal cortex BA9 were lysed in a radioimmunoprecipitation assay buffer and the extracted proteins were reduced, alkylated, de-lipidated, and digested with trypsin. Peptide concentrations were determined by Nanodrop (Thermo Fisher Scientific, Waltham, MA, USA) measurements at A280 nm. Nanoflow liquid chromatography-tandem mass spectrometry (LC-MS/MS) was performed using a nano-LC chromatography system, coupled online to a mass spectrometer through a nanospray ion source. LC-MS/MS data were acquired using XCalibur (Thermo Fisher Scientific) and the raw mass spectrometry data files were processed using MaxQuant (Max-Planck Institute of Biochemistry, Planegg, Germany). Peptide identifications were accepted in cases where they could be established at a probability >95.0%. MS/MS spectra were searched against the Swiss-Prot human database. In total, 4584 unique proteins were identified in all the samples.

### Statistical analysis

Statistical analyses were performed using the Statistical Package for the Social Sciences (SPSS version 26; IBM Corp., Armonk, NY, USA) and R software (version 3.6.1; R Foundation for Statistical Computing, Vienna, Austria). Regarding the demographic, clinical, and substance-related variables, the frequency data were analyzed using the Pearson’s chi-squared test. Group data were compared using Student’s *t* tests. When the data were non-normally distributed we used the Wilcoxon rank-sum test.

Regarding TBSS analysis with randomize algorithm, a *p* value < 0.05 after TFCE was considered significant. Given the known effects of age, sex, and years of education on FA^40–42^, these variables were added as covariates in both analyses along with head motion (estimated by eddy output). Within-group analysis also included the duration (years) of cocaine, nicotine, and alcohol use, as covariates. We used analysis of covariance (ANCOVA) to compare the global FA between the groups and we performed a linear regression analysis for within-group substance use associations.

Given that the TBSS cluster analysis revealed a major effect of years of crack-cocaine consumption on FA in the genu of the corpus callosum, and that previous studies demonstrated a main effect of cocaine in this anterior region^17^, we further explored this association by focusing on specific fibers of the frontal lobe. The anterior corpus callosum contains fibers responsible for prefrontal interhemispheric connectivity^43,44^. As structural abnormalities in the prefrontal cortex have been observed in patients with CUD^45,46^, we additionally selected the BA9 and BA8, as our main regions of interest (ROIs) for tractography^47^. These ROIs were extracted and projected into each participant’s surface through FreeSurfer software utilities. We reconstructed and analyzed the fibers connecting BA9 to BA8 in each hemisphere and fibers connecting the left to the right BA9.

Concerning the analysis of the WM bundles derived from tractography, we used ANCOVA to compare the groups considering sex and the duration of CU in life (years of CU/age).

For the proteomics analysis, the data were normalized and imputed. Moreover, differential expression analyses were performed in patients with CUD and HCs using protein-wise linear models combined with empirical Bayesian statistics implemented in the differential enrichment analysis of Proteomics data package and the limma package (R software), controlling for age, sex, PMI, and pH; *p* values < 0.05 and fold changes (FC) > 1.5 were considered significant. Gene set enrichment analysis was performed using Webgestalt software (access date: 20 April 2019). We required gene sets of 5–1000 genes per set to avoid specific or general annotations. We assessed pathways in two categories: GO-Cellular Component and KEGG pathways. Within each category, the terms/pathways with FDR < 0.05 were considered significant.

## Results

### Demographic and clinical characteristics

The cause of death was obtained from the medical examiner’s report and toxicological findings after death. The cause of death for individuals with CUD was cocaine overdose (*n* = 7) and cardiovascular disease (*n* = 1). The cause of death of the HCs was cardiovascular disease (*n* = 10) and pulmonary embolism (*n* = 2). The sociodemographic and clinical variables of the participants regarding the neuroimaging investigation are presented in Table 1. The data regarding the postmortem brain sample are presented in Table 1. Interestingly, the individuals in the CUD group had significantly higher age and less years of education compared to those in the HC group. Most of our samples consisted of severe cocaine users according with ASI-6. In addition, at least 1/3 of them could be classified as polysubstance users, as they met the criteria for cannabis, alcohol, or tobacco use disorders. There were no other statistically significant sociodemographic differences between the groups.

### TBSS comparison between the CUD and HC groups

**Fig. 1:**
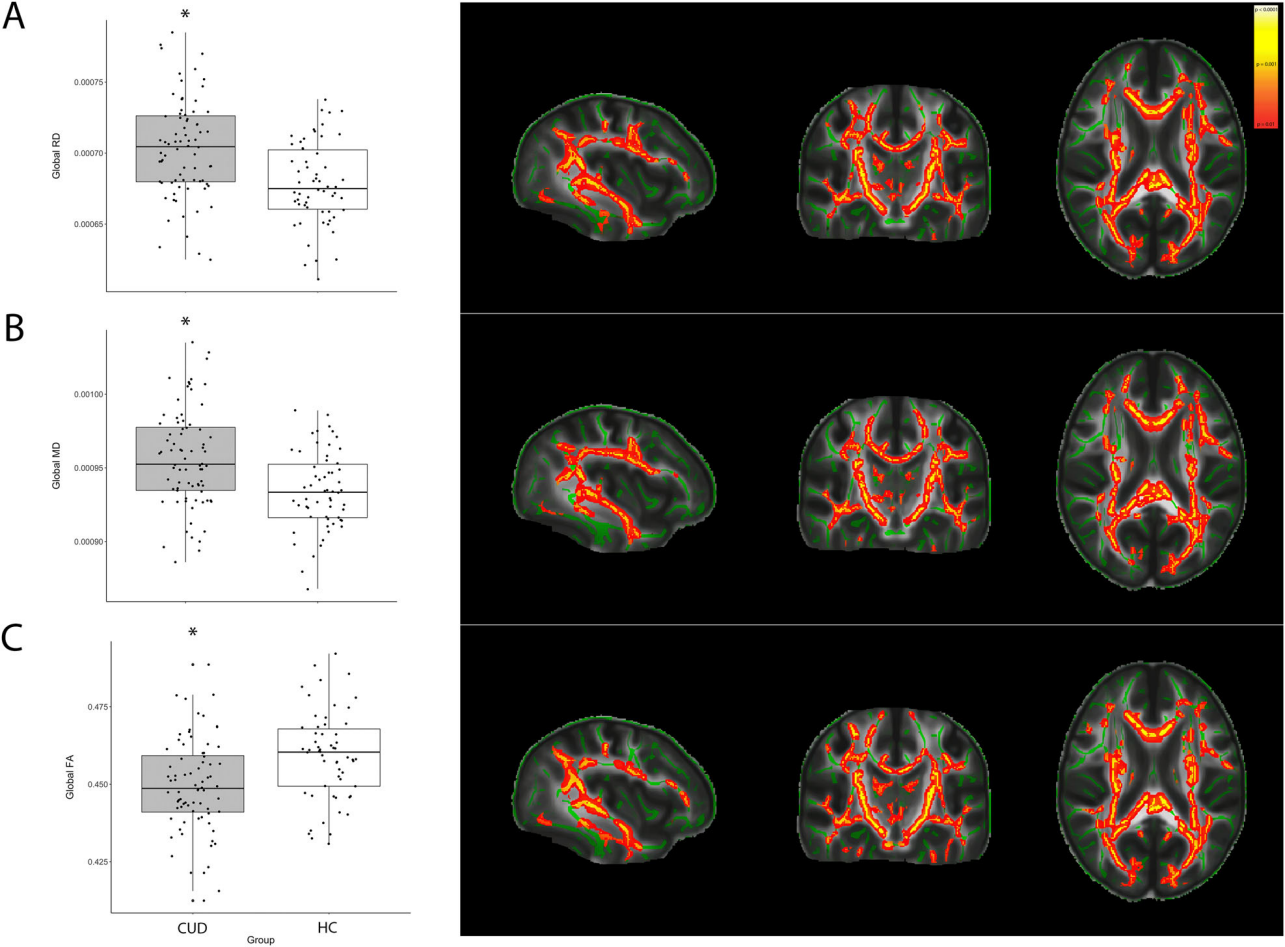
Boxplots for global DTI metrics in patients with CUD and HCs and the results from the TBSS analysis. Green line represents major white matter tracts projected on the average FA image; orange/red heat scale shows that the voxels with significant differences (*p* < 0.01, corrected for multiple comparisons) have different DTI metrics between the groups. Coronal view: *y* = 110; axial view: *z* = 92. **A** Higher RD values were observed in patients with CUD compared with HCs, both in the TBSS analysis and whole-brain average (*p* < 0.01). **B** Higher MD values were observed in patients with CUD compared with HCs, both in the TBSS analysis and whole-brain average (*p* < 0.01). **C** Lower FA values were observed in patients with CUD compared to HCs, both in the TBSS analysis and whole-brain average (*p* < 0.01). *CUD* cocaine use disorder, *HC* healthy control, *DTI* diffusion tensor imaging, *TBSS* tract-based special statistics, *RD* radial diffusion, *MD* mean diffusion, *FA* fractional anisotropy.

Compared with the HC group, the CUD group showed a significant decrease in the global FA (COD group: mean, 0.449 ± 0.015; HC group: mean, 0.459 ± 0.015; *p* < 0.001) and an increase in the global MD (COD group: mean, 0.00096 ± 3e-05; HC group: mean, 0.00093 ± 3e-05; *p* < 0.001) and RD (COD group: mean, 0.00070 ± 3e-05; HC group: mean, 0.00068 ± 3e-05; *p* < 0.001). The obtained results revealed that FA, RD, and MD alterations in the CUD group were widespread along the major WM tracts, as revealed by TBSS analysis (Fig. 1). Information on the percentage of significant voxels for each WM region can be found in Supplementary Table 1. There was also an independent effect of sex, with women having lower global FA and AD (both *p* < 0.001) values than men (TBSS showed a widespread difference in both metrics).

### TBSS within the CUD group

**Fig. 2:**
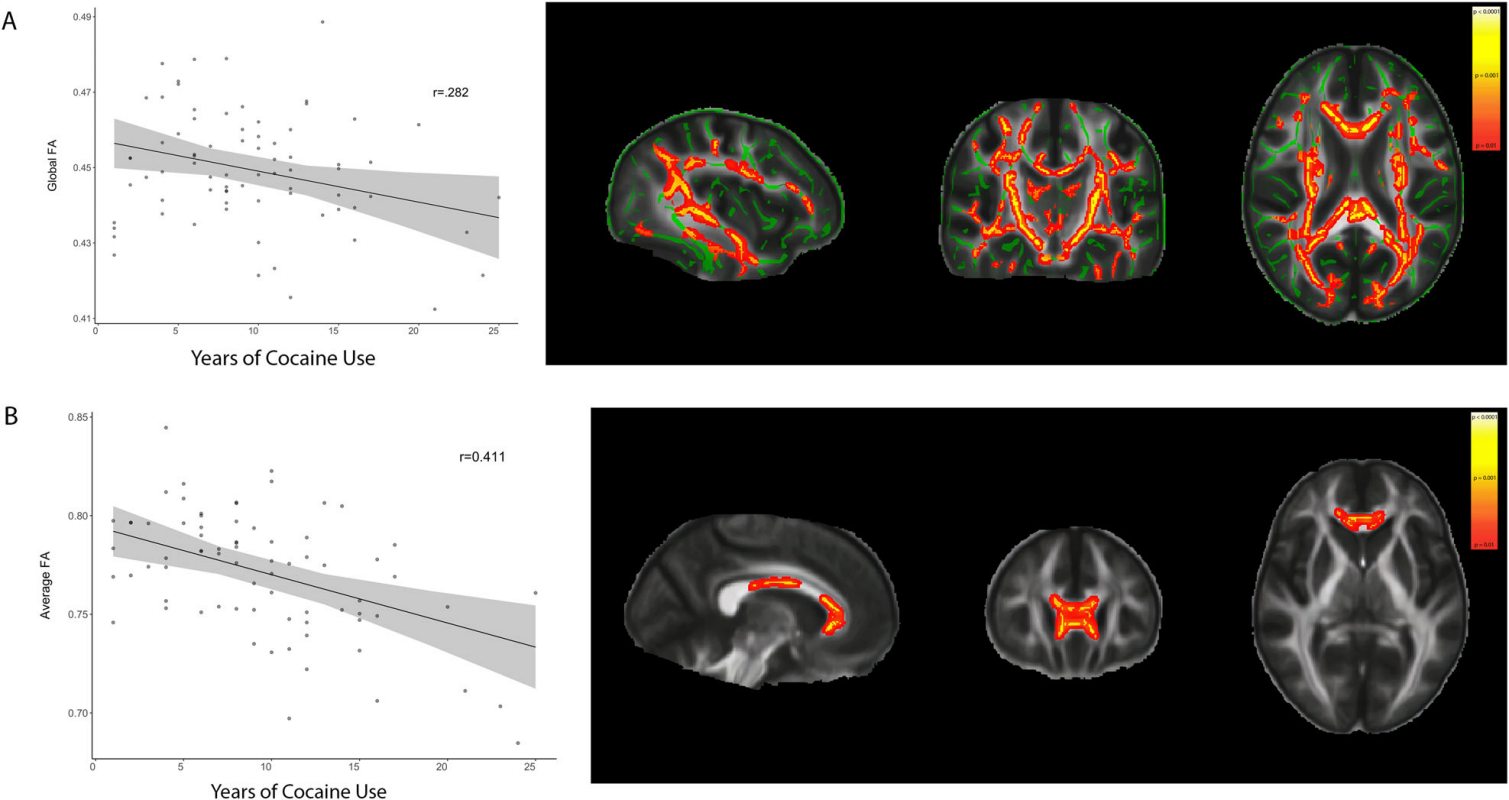
Years of crack-cocaine use and FA. **A** Association between years of crack-cocaine use and decrease in FA, as revealed by whole-brain average FA and TBSS analysis. Areas with the significant negative association in heat scale, the skeleton in green projected onto CUD’s average FA image. Sagittal view: *x* = 62; axial view: *z* = 86. **B** Main cluster comprising the genu and body of the corpus callosum, in which there was a strong negative association between the years of crack-cocaine use and FA. Center of mass at MNI coordinates: *x* = 94, *y* = 155, *z* = 81. *FA* fractional anisotropy, *TBSS* tract-based special statistics, *CUD* cocaine use disorder.

We performed an additional TBSS analysis within the CUD group to further characterize the alterations in DTI metrics. The results (*F*(6,68) = 2.693; *p* < 0.003; adjusted *R*^2^ = 0.137) showed that the global FA was negatively associated with the years of CU (*t* = −2.078; *p* = 0.0421) and female sex (*t* = −3.553; *p* < 0.001), but not with the years of alcohol or nicotine use. Cluster identification provided by TFCE revealed 12 FA clusters along multiple major WM tracks (including the cingulum bundle, corpus callosum, and uncinate fasciculus), in which the FA values were negatively correlated with years of CU (Fig. 2A). Most of these clusters were also negatively correlated with the female sex. One major cluster (Fig. 2B) comprising the genu and body of the corpus callosum (1505 voxels, center of mass: *x* = 94, *y* = 155, *z* = 81) showed an exclusive association with the years of CU (*t* = −3.145; *p* < 0.001) and no other variable (*F*(6,68) = 5.454; *p* = 0.001; adjusted *R*^2^ = 0.265).

**Fig. 3:**
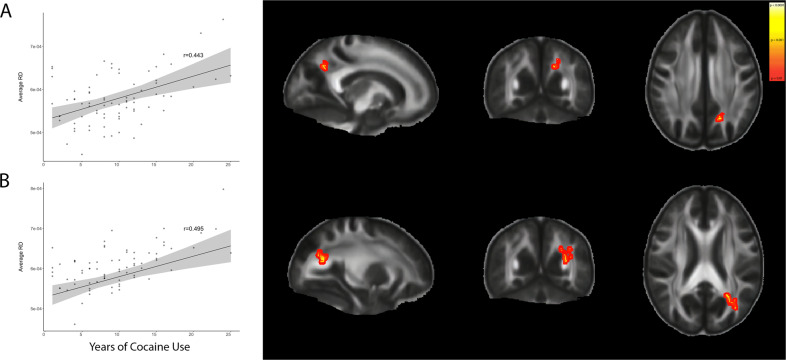
Clusters with a strong positive association between RD and years of crack-cocaine use. **A** Included regions of anterior thalamic radiation, cingulum, inferior fronto-occipital fasciculus, inferior longitudinal fasciculus, and superior longitudinal fasciculus. MNI coordinates are as follows: *x* = 106, *y* = 65, *z* = 107. **B** Included regions of anterior thalamic radiation, cingulum (cingulate gyrus), cingulum (hippocampus), forceps major; inferior fronto-occipital fasciculus, inferior longitudinal fasciculus, and superior longitudinal fasciculus. MNI coordinates are as follows: *x* = 117, *y* = 65, *z* = 95. *RD* radial diffusion.

The global RD showed no significant correlation with any studied variable, but two clusters (Fig. 3A, B) had RD values positively associated with the years of CU. The cluster displayed in Fig. 3A was related (*F*(6,68) = 4.428724; *p* < 0.001; adjusted *R*^2^ = 0.232) with years of cocaine consumption (*p* < 0.001; *t* = 4.073) and alcohol use (*p* = 0.028; *t* = 2.239). The cluster displayed in Fig. 3B was only related (*F*(6,68) = 6.587; *p* < 0.05; adjusted *R*^2^ = 0.319) with years of CU (*p* < 0.001; *t* = 4.699).

### Tractography of prefrontal fibers

Concerning the fibers connecting the left-BA9 to the right BA9, the CUD group presented lower FA (mean, 0.501 ± 0.041 vs. mean, 0.526 ± 0.030; *p* = 0.006) and higher RD (mean, 0.630 ± 0.065 vs. mean, 0.599 ± 0.046; *p* < 0.001) values compared with the HC group. Regarding the fibers connecting the left-BA9 to the left-BA8, the FA values were lower in the CUD group than in the HC group (mean, 0.385 ± 0.027 vs. mean, 0.395 ± 0.029; *p* = 0.026). Moreover, we found lower FA (mean, 0.405 ± 0.027 vs. mean, 0.414 ± 0.026; *p* = 0.046) and higher RD (mean, 0.631 ± 0.038 vs. mean, 0.6179 ± 0.0338; *p* = 0.044) values in the CUD group when comparing tracts connecting the right BA9 to the right BA8.

### Tractography clinical associations

**Fig. 4:**
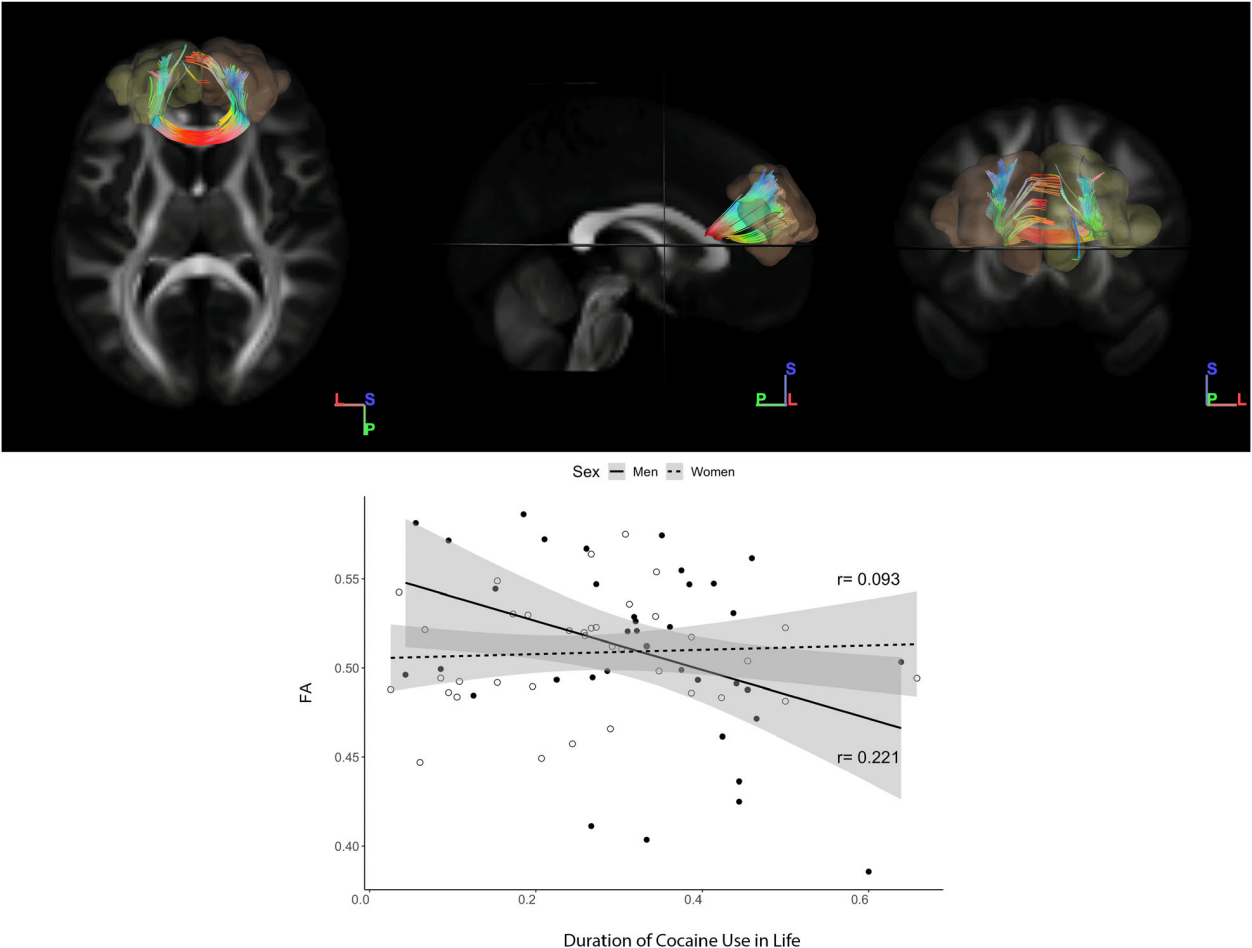
White matter bundle connecting left to right BA9. Colors are respecting the direction of the fibers. Graphs show the associations between the tract average FA and MD values with the years of cocaine use considering the interaction effect of sex. *MD* mean diffusion, *FA* fractional anisotropy, *BA9* Brodmann area 9.

An interaction effect of years of crack-CU and sex within the CUD group was observed on WM tracts connecting the left-BA9 to the right BA9 for FA (*F*(5,69) = 2.51; *p* = 0.037; adjusted *R*^2^ = 0.093). Specifically, we found a negative association between the CU duration in life and FA values in this tract, only in the male users (*t* = −2.394; *p* = 0.019; Fig. 4).

### Proteomic analysis results

Based on the effect of the cocaine consumption history on the prefrontal fibers, and concerning the evidence obtained from previous studies regarding the alterations in WM integrity in the prefrontal cortex^18,45^, proteomic analyses were performed in the BA9 postmortem brain tissue. We used two sets of criteria to define significantly differentially expressed proteins, based on the raw and the adjusted *p* value, respectively. In both scenarios, we combined the *p* value threshold with log2 (FC). By setting the threshold as a *p* value < 0.05 and |logFC|>log2 (1.5), we identified 87 proteins that were significantly differentially expressed between the CUD and the HC group. The 87 proteins were used for the enrichment analysis. Pathways related to secretory vesicle formation and trafficking, focal adhesion, and cell-substrate junction were enriched in the CUD group (Supplementary Table 2), in line with previous human myelin proteome analysis findings^48^.

**Fig. 5:**
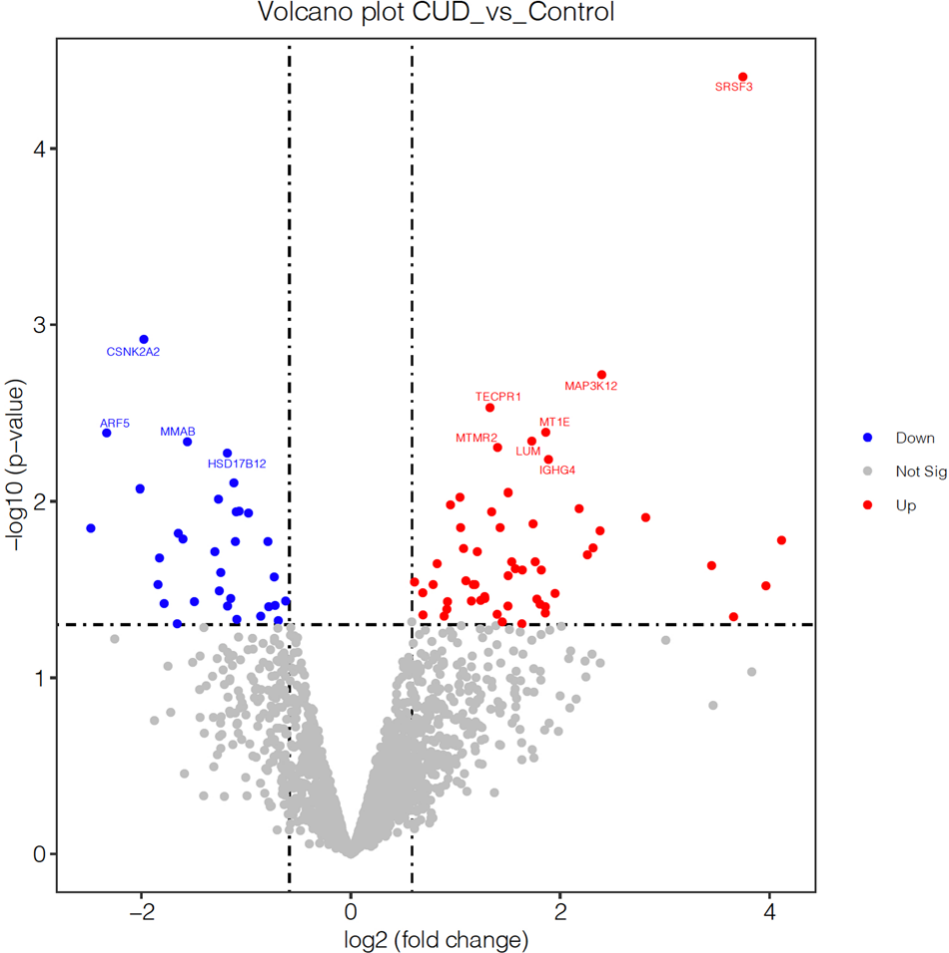
Differentially expressed proteins in patients with CUD and HCs. Volcano plot shows the differentially expressed proteins in patients with CUD and HCs. *CUD* cocaine use disorder, *HC* healthy control.

Further analysis with a threshold of *p* < 0.05 and |logFC| >log2(1.5) reduced the number of significantly differentially expressed proteins to 11 (Supplementary Table 3). As depicted in the volcano plot (Fig. 5), in the CUD group, we found reduced levels in the following proteins: casein kinase 2 subunit alpha serine-threonine kinase, involved in controlling synapse organization and stability by regulation of NMDA subunits^49^; ADP Ribosylation Factor 5 (ARF5), an activator of phospholipase D^50^, which is critically involved in myelin development^51^; and Metabolism of Cobalamin Associated B (MMAB) that catalyzes vitamin B12^52^, which is known to have a key role in myelin repair and maintenance^53^. In addition, among the proteins with higher expression in CUD are serine and arginine rich splicing factor 3 (SRSF3), involved in regulating innate immune responses in activated microglia^54^; Lumican (LUM), which has an important role in innate immunity^55^; and Myotubularin-related protein 2 (MTMR2), a protein involved in membrane trafficking via dephosphorylation of phosphoinositides, such as PI(3)P. The mass spectrometry proteomics data have been deposited to the ProteomeXchange Consortium via the PRIDE partner repository with the data set identifier PXD025269.

## Discussion

In this study, we showed that crack-cocaine users presented globally distributed alterations in DTI measures (lower FA, higher MD, and RD values) compared with HCs. Moreover, we found that the FA values decreased with increasing years of crack-CU and that this association is particularly strong in a large cluster in the genu and body of the corpus callosum. This area is responsible for the interhemispheric connection of the prefrontal cortex. By reconstructing the specific WM bundles passing through this cluster, connecting both BA9 hemispheres, we found an interaction effect, in which male users with longer CU histories had lower FA and higher RD values. Finally, we found that altered levels of proteins are important for WM integrity and neuroinflammation in the dorsolateral prefrontal cortex BA9 of postmortem brains of patients with CUD. The latter supported the notion that altered DTI metrics maybe because of the impaired WM integrity, perhaps caused by alterations in synapse organization and stability and by vesicular trafficking and, therefore, they are not just a result of non-pathological microstructural disarrangements in the WM, which would also cause altered FA values^10^.

Regarding the development of substance use disorders, altered prefrontal cortex structure/functioning has been implicated in the transition from goal-directed to habitual drug-seeking behavior, and finally to compulsive patterns of drug use^56^. In this sense, although some previous DTI studies have indicated an association between CUD and altered WM integrity in the frontal areas and in the anterior corpus callosum^19,57^, our findings are novel, as we showed that chronic smoked CU is progressively implicated in decreasing the WM in intrahemispheric connections of the BA9.

We also found two smaller clusters spanning through the dorsal cingulum and posterior thalamic radiation, which presented a strong positive association between RD and years of CU. The cingulum has an important role in connecting the structures of the limbic system, such as the hippocampus and cingulate gyrus. A previous study has supported its role in executive control, emotion, pain, and episodic memory. Especially, the association of pain and episodic memory with the dorsal cingulum and many DTI alterations have been reported in these regions in patients with psychiatric and neurological disorders^58^. RD increases in parietal regions were shown to be associated with poor decision making in patients with CUD^20^.

To our knowledge, this was the first DTI study that reported significant whole-brain differences between patients with CUD and HCs concerning FA, RD, and MD. These results reflected a widespread effect of cocaine dependence in the brain, in contrast to the more region-specific effects found in previous studies^59,60^. This might be because several previous studies have focused on snorted cocaine with less attention being paid to polysubstance use, which ultimately may result in participants with less-severe substance use disorder-induced toxicity. This idea is corroborated by findings showing that altered global FA, MD, and RD were associated with numerous additionally abused substances in patients with CUD^45^. Global FA and RD values were also associated with years of CU in patients with comorbid CUD and alcohol use disorder^18^. Given that the majority of cocaine users may use and abuse additional drugs^61^, including polysubstance drugs, the inclusion of patients with CUD should allow better generalization of the current findings to real-world settings.

Moreover, previous studies have not specified or not focused on users of smoked cocaine. However, the route of administration and/or the presence of adulterants^62^ related to “crack” cocaine could play an important role in the neurotoxic effect of the drug, as it presents different and more detrimental consequences in the organism^63^. These different effects reflect on clinical measures, where snorted users may achieve better treatment outcomes and better neuropsychological profile than smokers^63^, particularly in prefrontal cortex-dependent tasks^64^.

In this study, we found an interaction between the length of CU in life and sex. We found that higher CU duration in life was associated with lower FA values in male participants. This was an interesting result, as studies have shown strong sex differences regarding clinical patterns of substance use disorders, especially in CUD^33^. Men outnumber women in a proportion of 3:1 in CUD prevalence^65^; nevertheless, women are more likely to develop CUD after the first use^66^, progress faster from recreational to pathological use^67^, relapse after treatment^68^, and present a more severe CUD^33^. Many studies on DTI investigated one sex only^15,45^ or had an uneven distribution of sexes^12,60,69^, which might have masked any possible sex interaction.

Although in our study, there was a significant association between lifelong CU duration and FA in the tract connecting the BA9s, women had lower global FA values than men in both the CUD and HC groups, except for in the inter-BA9 tract. There are well-documented sexual dimorphisms in DTI studies of healthy participants, the direction of this effect is somewhat conflicting. Nevertheless, a review of the effect of sex on brain connectivity reported that women had overall lower FA values than men^70^. Regarding our findings, this sex effect was also present when analyzing the TBSS AD results, with women also showing lower global AD values. As FA is a compound metric, which relies on the diffusion ratio along the main axis with respect to the diffusion as a whole, there may be multiple paths that mediate the decrease in FA. There could be an increase in *λ*2 and *λ*3 (i.e., higher diffusion perpendicular to the main axis, interpreted as RD), or a decrease in *λ*1 (i.e., lower diffusion in the main axis, interpreted as AD). Increases in RD^9^ have been consistently reported as a marker for demyelination and decreases in AD are not well established in the literature, the precise neurobiological interpretation of FA, RD, and MD is hard to establish in areas of crossing fibers^71^. In this study, FA differences owing to sex effects regardless of the group were usually accompanied by a decrease in AD in women; in line, the decrease in FA because of CUD was usually accompanied by an increase in RD, suggesting in vivo evidence of WM deficits associated with CU.

We also found ex vivo evidences of WM deficits associated with CUD. Our postmortem brain proteomics approach revealed evidence of WM deficits and neuroinflammation associated with CUD. To our knowledge, our study was the first that performed an in-depth proteomic analysis in brain samples of participants with CUD, and the first to report a decrease in myelination-related proteins in the prefrontal cortex. Interestingly, we found significantly decreased levels of ARF5 (i.e., an activator of phospholipase D)^50^. Specifically, phospholipase D4 has an important role in the mechanisms of myelin development^51^. Furthermore, ARF5 was identified as part of the myelin proteome in the central and peripheral nervous systems^72,73^, suggesting that low ARF5 levels have implications for WM integrity. Reduced levels of MMAB were also found in the CUD group. MMAB has a key role in vitamin B12 metabolism, whereas vitamin B12 deficiencies are critically associated with myelin-related disorders, such as multiple sclerosis^74^. Vitamin B12 is also required to produce methionine, which is necessary for methylation reactions that are essential for myelin maintenance and repair^53^. Evidence suggests that reduced vitamin B12 and methionine levels result in demyelination^75^. In contrast, we detected an increase in the MTMR2 levels in CUD brains, which could be related to a compensatory mechanism. Disruption of MTMR2 is associated with myelin abnormalities, as it may regulate the membrane transport, which is crucially important in the neurons and Schwann cells^76^.

We also observed reduced levels of CSNK2A2, which is part of the kinase CK2 group of proteins involved in neurodevelopment and synaptic plasticity^77^. However, more recently, CSNK2A2 was found to regulate neuroimmune responses through Th17 cell infiltration into the brain in a rodent model of autoimmune encephalomyelitis^78^. This is relevant considering the upregulation of LUM and SRSF3 found in the CUD group. Increased LUM levels are associated with Th17 cell activation and inflammation^79^. Moreover, activated microglia cells highly express SRSF3 to regulate inflammatory gene expression and translation^54^. SRSF3 has several binding sites to transcripts of upregulated innate immune genes. This suggests that an imbalance in the levels of immune mediators, such as CSNK2A2, LUM, and SRSF3, could lead to neuroinflammatory signaling cascade in the prefrontal cortex. Given that neuroinflammation is a primary hallmark in several myelin-related disorders and since a misguided inflammatory response is often implicated in demyelination^80^, we found evidence that supported the association of these conditions with chronic substance use throughout life.

These findings from our proteomics analysis could corroborate the observed increase in RD, as a marker of demyelination. A previous postmortem brain study found a decrease in the expression of myelin-related genes in the nucleus accumbens of cocaine abusers^81^. Interestingly, the authors suggested a dysregulation of myelination in these participants. Finally, the negative association between FA and age was also accompanied by an increase in RD and no change in AD, indicating that the mechanism through which FA decreases in patients with CUD is more related to aging compared with the effect of sex.

Nevertheless, this study had some limitations that should be acknowledged. First, the study design did not allow us to infer whether the DWI alterations are the cause or consequence of CUD. Second, although the DTI and postmortem findings indicate cognitive disruptions that should be reflected in impaired decision-making, affective control, and impulsivity, the present results do not include assessments of psychometric or cognitive/laboratory tests of these constructs. Additional studies would help confirm these hypotheses by including representative assessments in the test batteries and examining their associations with neuroimaging and histology outcomes. In addition, DWI metrics such as FA, RD, and MD are dependent on multiple uncontrolled factors, such as axonal coherence, membrane permeability, and fiber orientation^10^ and are especially challenging to analyze in crossing fibers regions, which comprises one-third of the brain^71^. Changes in these metrics must be interpreted carefully, and must not be acknowledged as a synonym of changes in “WM integrity”. Finally, despite using two different approaches (in vivo brain imaging and ex vivo brain proteomic analysis) converging to the idea of WM impairment in CUD, we acknowledge that one study assessed data from smoked cocaine users in southern Brazil, whereas the other included both smoked and snorted cocaine users from Houston, TX, USA. Therefore, we could not rule out potential cultural, social, and even ancestry effects in this study.

Despite these limitations, we believe that we demonstrated convergent evidence from in vivo DTI findings and ex vivo proteomic analysis of WM impairments in patients with CUD. Considering that CUD treatment usually has poor outcomes and high relapse rates^82^, such findings bring new insights into sex differences and their pathophysiology.

## Supplementary information

Supplementary Methods

Supplementary Table 1

Supplementary Table 2

Supplementary Table 3
