# Supplementary material for: White matter deficits in cocaine use disorder: convergent evidence from in vivo diffusion tensor imaging and ex vivo proteomic analysis
Source: Transl Psychiatry. 2021 Apr 29;11:252. doi: 10.1038/s41398-021-01367-x (PMC8081729; doi:10.1038/s41398-021-01367-x)
Supplement: Supplementary file 1 — Supplementary Methods [file 41398_2021_1367_MOESM1_ESM.docx]

**White matter deficits in cocaine use disorder: Convergent evidence from *in vivo* diffusion tensor imaging and *ex vivo* proteomic analysis**

***Supplemental Methods***

**Image acquisition**

Brain images were acquired on a 3T Signa GE scanner (GE Healthcare, Chicago, IL, USA) with an eight-channel head coil at the Brain Institute of Rio Grande do Sul, Brazil. At the beginning of the scanning session, a single, high-resolution volumetric T1-weighted anatomic image was collected (echo time, 2.18 ms; repetition time, 6.1 ms; flip angle, 11°; number of excitations, 1; slice thickness, 1 mm; field of view, 256 mm; resolution, 256×256). Whole-brain DWI was performed in the transverse plane using a single-shot diffusion sensitized spin echo-planar imaging sequence, using the following parameters: b-factor, 750 s/mm^2^; one b0 image and 33 DWI with gradient directions; TR, 13000 ms; TE, 81.1 ms; slice thickness, 2.4; slice gap, 0; flip angle, 90; acquisition matrix, 120×120.

**TBSS’ processing**

DWIs were brain-extracted and corrected for eddy-current distortions using FMRIB Software Library (FSL; Oxford, UK) version 6.0 tools, followed by a visual quality assessment. The FA, MD, AD, and RD maps were calculated from the DTI parameters using DTIFit in FSL. AD was derived as the eigenvalue of the main diffusion direction ($\lambda$1) output from DTIFit . RD was calculated as the average between $\lambda$2 and $\lambda$3.

Voxelwise statistical analysis of the FA data was performed using TBSS, ^1^ which is part of FSL ^2^. All participants were registered to the standard MNI152 space using the FSL's nonlinear registration with the FMRIB58_FA template image. Then, TBSS projected all participants’ FA data onto a mean FA tract skeleton using a threshold value of FA>0.20. This process was performed in all participants for between-group comparisons (crack-cocaine users vs HCs) and in the cocaine group for within-group comparisons. Then, voxelwise cross-participant statistical analysis was performed between the groups using FSL's general linear model software randomize*.* The results were corrected with a threshold free cluster enhancement approach (TFCE) ^3^. MD, RD, and AD values were also analyzed with TBSS, with each parameter being projected onto the FA skeleton for an independent analysis.

Additionally, whole-brain metrics (global FA, RD, MD, and AD) were calculated by averaging all non-zero voxels of each participant within the skeleton mask. Finally, masks based on the JHU ICBM DTI 81 White Matter Atlas ^4^ were used to extract the regions of interest (ROI), which contained significant voxels in the TBSS analyses. A percentage of significant voxels for each WM region was computed by dividing the number of voxels with an intensity >0.95 (i.e., p-value<0.05) of the randomized output image by the total number of non-zero voxels in the FA skeleton of that region.

**Tractography processing and analysis**

Considering TBSS’ methodological limitations, especially the intraparticipant variability for minor tracts and anatomical specificity of tracts within the skeleton, we realized that this method lacks tract specificity in regions where fiber bundles originating from multiple brain region merge, such as the superior projections of the corpus callosum and the corona radiata ^5^. As an alternative, we also conducted a tractography-based analysis in each participant’s native space to investigate the connectivity of the cortical regions whose specific tracts passed through the affected WM regions in TBSS. For this analysis, the T1-weighted anatomical dataset was processed with FreeSurfer version 6.0.0 (Martinos Center for Biomedical Imaging, Charlestown, MA, USA). Recon-all pipeline was performed, which included brain extraction, intensity normalization, and cortical parcellation ^6-8^. DSI Studio (<http://dsi-studio.labsolver.org/>) software was used for tractography. The diffusion data for each participant were reconstructed in the corresponding T1 image and, therefore, the fiber tracking could be performed in the participants’ native space. Freesurfer’s parcellation was used to account for individual differences. Each participant's DTI was registered in T1-space, following one of the DSI-studio pipelines (<http://dsi-studio.labsolver.org/Manual/tract-specific-analysis>). The rotation of the b-table was performed automatically together with the registration. The b-table was checked by an automatic quality control routine to ensure its accuracy ^9^. The diffusion tensor was calculated and the ROIs were extracted and projected into each participant’s surface trough FreeSurfer utilities. A deterministic fiber tracking algorithm was used ^10^. The FA threshold was 0.121875 (standard value, DSI studio) and the angular threshold was randomly selected from 15 to 90 degrees. The step size was randomly selected from 0.5 to 1.5 voxels. The fiber trajectories were smoothed by averaging the propagation direction with a percentage of the previous direction. The percentage was randomly selected from 0% to 95%. Tracks with length <30 or >100 mm were discarded. In total, 5000 streamlines were calculated. Topology-informed pruning was applied to the tractography with one iteration to remove false connections ^10^. All tracts were visually inspected. The mean DTI metrics of the tract were extracted for each participant.

1. Smith SM, Jenkinson M, Johansen-Berg H, Rueckert D, Nichols TE, Mackay CE *et al.* Tract-based spatial statistics: voxelwise analysis of multi-subject diffusion data. *Neuroimage* 2006; **31**(4)**:** 1487-1505.

2. Smith SM, Jenkinson M, Woolrich MW, Beckmann CF, Behrens TE, Johansen-Berg H *et al.* Advances in functional and structural MR image analysis and implementation as FSL. *Neuroimage* 2004; **23 Suppl 1:** S208-219.

3. Smith SM, Nichols TE. Threshold-free cluster enhancement: addressing problems of smoothing, threshold dependence and localisation in cluster inference. *Neuroimage* 2009; **44**(1)**:** 83-98.

4. Mori S, van Zijl P, Nagae-Poetscher L. *MRI Atlas of Human White Matter 1st Edition*. Elsevier  Science2005.

5. Bach M, Laun FB, Leemans A, Tax CM, Biessels GJ, Stieltjes B *et al.* Methodological considerations on tract-based spatial statistics (TBSS). *Neuroimage* 2014; **100:** 358-369.

6. Dale AM, Fischl B, Sereno MI. Cortical surface-based analysis. I. Segmentation and surface reconstruction. *Neuroimage* 1999; **9**(2)**:** 179-194.

7. Fischl B, Dale AM. Measuring the thickness of the human cerebral cortex from magnetic resonance images. *Proc Natl Acad Sci U S A* 2000; **97**(20)**:** 11050-11055.

8. Fischl B, Liu A, Dale AM. Automated manifold surgery: constructing geometrically accurate and topologically correct models of the human cerebral cortex. *IEEE Trans Med Imaging* 2001; **20**(1)**:** 70-80.

9. Schilling KG, Yeh FC, Nath V, Hansen C, Williams O, Resnick S *et al.* A fiber coherence index for quality control of B-table orientation in diffusion MRI scans. *Magn Reson Imaging* 2019; **58:** 82-89.

10. Yeh FC, Panesar S, Barrios J, Fernandes D, Abhinav K, Meola A *et al.* Automatic Removal of False Connections in Diffusion MRI Tractography Using Topology-Informed Pruning (TIP). *Neurotherapeutics* 2019; **16**(1)**:** 52-58.
