# Supplementary material for: White matter deficits in cocaine use disorder: convergent evidence from in vivo diffusion tensor imaging and ex vivo proteomic analysis
Source: Transl Psychiatry. 2021 Apr 29;11:252. doi: 10.1038/s41398-021-01367-x (PMC8081729; doi:10.1038/s41398-021-01367-x)
Supplement: Supplementary file 2 — Supplementary Table 1 [file 41398_2021_1367_MOESM2_ESM.docx]

Table S1. Percentage of significant voxels for each white matter region

| **Region** | **Total Number of Voxels** | **FA Voxels** | **FA %** | **RD Voxels** | **RD %** | **MD Voxels** | **MD Voxels %** |
| --- | --- | --- | --- | --- | --- | --- | --- |
| Middle cerebellar peduncle | 899.0 | 548.0 | 61.0 | 460.0 | 51.2 | 280.0 | 31.1 |
| Pontine crossing tract a part of MCP | 289.0 | 191.0 | 66.1 | 192.0 | 66.4 | 186.0 | 64.4 |
| Genu of corpus callosum | 1798.0 | 1249.0 | 69.5 | 1311.0 | 72.9 | 1261.0 | 70.1 |
| Body of corpus callosum | 3177.0 | 1833.0 | 57.7 | 2051.0 | 64.6 | 2017.0 | 63.5 |
| Splenium of corpus callosum | 2669.0 | 1372.0 | 51.4 | 1389.0 | 52.0 | 1329.0 | 49.8 |
| Fornix column and body of fornix | 154.0 | 23.0 | 14.9 | 0.0 | 0.0 | 0.0 | 0.0 |
| Corticospinal tract R | 261.0 | 189.0 | 72.4 | 194.0 | 74.3 | 162.0 | 62.1 |
| Corticospinal tract L | 285.0 | 139.0 | 48.8 | 149.0 | 52.3 | 113.0 | 39.6 |
| Medial lemniscus R | 95.0 | 78.0 | 82.1 | 89.0 | 93.7 | 86.0 | 90.5 |
| Medial lemniscus L | 100.0 | 86.0 | 86.0 | 90.0 | 90.0 | 89.0 | 89.0 |
| Inferior cerebellar peduncle R | 96.0 | 78.0 | 81.3 | 91.0 | 94.8 | 0.0 | 0.0 |
| Inferior cerebellar peduncle L | 93.0 | 55.0 | 59.1 | 74.0 | 79.6 | 75.0 | 80.6 |
| Superior cerebellar peduncle R | 208.0 | 195.0 | 93.8 | 181.0 | 87.0 | 102.0 | 49.0 |
| Superior cerebellar peduncle L | 193.0 | 176.0 | 91.2 | 164.0 | 85.0 | 120.0 | 62.2 |
| Cerebral peduncle R | 577.0 | 461.0 | 79.9 | 465.0 | 80.6 | 367.0 | 63.6 |
| Cerebral peduncle L | 597.0 | 482.0 | 80.7 | 455.0 | 76.2 | 304.0 | 50.9 |
| Anterior limb of internal capsule R | 771.0 | 303.0 | 39.3 | 253.0 | 32.8 | 108.0 | 14.0 |
| Anterior limb of internal capsule L | 766.0 | 410.0 | 53.5 | 376.0 | 49.1 | 259.0 | 33.8 |
| Posterior limb of internal capsule R | 899.0 | 681.0 | 75.8 | 619.0 | 68.9 | 466.0 | 51.8 |
| Posterior limb of internal capsule L | 895.0 | 700.0 | 78.2 | 684.0 | 76.4 | 503.0 | 56.2 |
| Retrolenticular part of internal capsule R | 671.0 | 371.0 | 55.3 | 377.0 | 56.2 | 306.0 | 45.6 |
| Retrolenticular part of internal capsule L | 720.0 | 570.0 | 79.2 | 554.0 | 76.9 | 415.0 | 57.6 |
| Anterior corona radiata R | 1491.0 | 807.0 | 54.1 | 966.0 | 64.8 | 943.0 | 63.2 |
| Anterior corona radiata L | 1551.0 | 1140.0 | 73.5 | 1215.0 | 78.3 | 1036.0 | 66.8 |
| Superior corona radiata R | 1418.0 | 778.0 | 54.9 | 853.0 | 60.2 | 601.0 | 42.4 |
| Superior corona radiata L | 1369.0 | 820.0 | 59.9 | 838.0 | 61.2 | 648.0 | 47.3 |
| Posterior corona radiata R | 788.0 | 519.0 | 65.9 | 596.0 | 75.6 | 569.0 | 72.2 |
| Posterior corona radiata L | 743.0 | 371.0 | 49.9 | 512.0 | 68.9 | 551.0 | 74.2 |
| Posterior thalamic radiation R | 1197.0 | 781.0 | 65.2 | 734.0 | 61.3 | 520.0 | 43.4 |
| Posterior thalamic radiation L | 1062.0 | 697.0 | 65.6 | 677.0 | 63.7 | 499.0 | 47.0 |
| Sagittal stratum R | 571.0 | 346.0 | 60.6 | 415.0 | 72.7 | 396.0 | 69.4 |
| Sagittal stratum L | 477.0 | 263.0 | 55.1 | 295.0 | 61.8 | 269.0 | 56.4 |
| External capsule R | 1318.0 | 737.0 | 55.9 | 732.0 | 55.5 | 642.0 | 48.7 |
| External capsule L | 1398.0 | 745.0 | 53.3 | 791.0 | 56.6 | 698.0 | 49.9 |
| Cingulum cingulate gyrus R | 416.0 | 125.0 | 30.0 | 163.0 | 39.2 | 207.0 | 49.8 |
| Cingulum cingulate gyrus L | 433.0 | 10.0 | 2.3 | 27.0 | 6.2 | 39.0 | 9.0 |
| Cingulum hippocampus R | 242.0 | 129.0 | 53.3 | 119.0 | 49.2 | 204.0 | 84.3 |
| Cingulum hippocampus L | 222.0 | 70.0 | 31.5 | 69.0 | 31.1 | 0.0 | 0.0 |
| Fornix Stria terminalis R | 325.0 | 178.0 | 54.8 | 190.0 | 58.5 | 139.0 | 42.8 |
| Fornix Stria terminalis L | 357.0 | 219.0 | 61.3 | 173.0 | 48.5 | 72.0 | 20.2 |
| Superior longitudinal fasciculus R | 1463.0 | 733.0 | 50.1 | 822.0 | 56.2 | 686.0 | 46.9 |
| Superior longitudinal fasciculus L | 1414.0 | 710.0 | 50.2 | 834.0 | 59.0 | 879.0 | 62.2 |
| Superior fronto-occipital fasciculus R | 92.0 | 23.0 | 25.0 | 7.0 | 7.6 | 5.0 | 5.4 |
| Superior fronto-occipital fasciculus L | 97.0 | 21.0 | 21.6 | 25.0 | 25.8 | 39.0 | 40.2 |
| Uncinate fasciculus R | 77.0 | 40.0 | 51.9 | 48.0 | 62.3 | 41.0 | 53.2 |
| Uncinate fasciculus L | 75.0 | 63.0 | 84.0 | 67.0 | 89.3 | 55.0 | 73.3 |
| Tapetum R | 25.0 | 21.0 | 84.0 | 23.0 | 92.0 | 17.0 | 68.0 |
| Tapetum L | 0.0 | 0.0 |  | 0.0 | 0.0 | 0.0 |  |
