# Supplementary material for: White matter deficits in cocaine use disorder: convergent evidence from in vivo diffusion tensor imaging and ex vivo proteomic analysis
Source: Transl Psychiatry. 2021 Apr 29;11:252. doi: 10.1038/s41398-021-01367-x (PMC8081729; doi:10.1038/s41398-021-01367-x)
Supplement: Supplementary file 3 — Supplementary Table 2 [file 41398_2021_1367_MOESM3_ESM.docx]

| **Supplementary Table 2**. The top enriched GO-CC terms | | | | | | | |
| --- | --- | --- | --- | --- | --- | --- | --- |
| **Category** | **ID** | **GO term or pathway** | **# genes in term/pathway** | **# overlapped genes** | ***p*-value** | **FDR** | **Symbols of overlapped genes** |
| CC | GO:0005912 | adherens junction | 538 | 14 | 3.10E-08 | 1.95E-05 | *RPL30, CXADR, IQGAP1, ANXA2, PPP1CC, SCARB2, CAMSAP3, TLN1, ITGAV, RAB21, LPP, ANXA1, MYH9, ACTB* |
| CC | GO:0070161 | anchoring junction | 554 | 14 | 4.46E-08 | 1.95E-05 | *RPL30, CXADR, IQGAP1, ANXA2, PPP1CC, SCARB2, CAMSAP3, TLN1, ITGAV, RAB21, LPP, ANXA1, MYH9, ACTB* |
| CC | GO:0099503 | secretory vesicle | 976 | 18 | 5.09E-08 | 1.95E-05 | *CSNK2A2, MTMR2, CXADR, BRCA2, A2M, TMEM163, ROGDI, IQGAP1, MT3, ANXA2, PNP, RAB8A, APOOL, ITGAV, CAB39, TXNDC5, CPNE1, TTR* |
| CC | GO:0005925 | focal adhesion | 404 | 11 | 7.03E-07 | 1.59E-04 | *RPL30, IQGAP1, PPP1CC, SCARB2, TLN1, ITGAV, RAB21, LPP, ANXA1, MYH9, ACTB* |
| CC | GO:0005924 | cell-substrate adherens junction | 407 | 11 | 7.56E-07 | 1.59E-04 | *RPL30, IQGAP1, PPP1CC, SCARB2, TLN1, ITGAV, RAB21, LPP, ANXA1, MYH9, ACTB* |
| CC | GO:0030055 | cell-substrate junction | 411 | 11 | 8.32E-07 | 1.59E-04 | *RPL30, IQGAP1, PPP1CC, SCARB2, TLN1, ITGAV, RAB21, LPP, ANXA1, MYH9, ACTB* |
| CC | GO:0005775 | vacuolar lumen | 170 | 7 | 6.04E-06 | 9.89E-04 | *LUM, PRELP, ANXA2, SCARB2, BGN, TXNDC5, TTR* |
| CC | GO:0005773 | vacuole | 760 | 13 | 1.10E-05 | 0.0016 | *TECPR1, LUM, MTMR2, PIP4K2C, PRELP, ANXA2, SCARB2, RDH14, BGN, ANXA1, TXNDC5, CPNE1, TTR* |
| CC | GO:0044437 | vacuolar part | 552 | 11 | 1.40E-05 | 0.0018 | *TECPR1, LUM, MTMR2, PRELP, ANXA2, SCARB2, RDH14, BGN, TXNDC5, CPNE1, TTR* |
| CC | GO:0030133 | transport vesicle | 374 | 9 | 2.10E-05 | 0.0024 | *MTMR2, PEF1, TMEM163, ROGDI, MT3, RAB8A, BGN, SEC23A, ARFGEF3* |
| CC | GO:0030141 | secretory granule | 831 | 13 | 2.83E-05 | 0.0030 | *CSNK2A2, CXADR, BRCA2, A2M, IQGAP1, ANXA2, PNP, APOOL, ITGAV, CAB39, TXNDC5, CPNE1, TTR* |
| CC | GO:0043202 | lysosomal lumen | 94 | 5 | 4.13E-05 | 0.0039 | *LUM, PRELP, SCARB2, BGN, TXNDC5* |
| CC | GO:0001726 | ruffle | 169 | 6 | 6.65E-05 | 0.0059 | *IQGAP1, ANXA2, TLN1, ITGAV, MYH9, NME1* |
| CC | GO:0005764 | lysosome | 669 | 11 | 8.10E-05 | 0.0063 | *TECPR1, LUM, PRELP, ANXA2, SCARB2, RDH14, BGN, ANXA1, TXNDC5, CPNE1, TTR* |
| CC | GO:0000323 | lytic vacuole | 670 | 11 | 8.20E-05 | 0.0063 | *TECPR1, LUM, PRELP, ANXA2, SCARB2, RDH14, BGN, ANXA1, TXNDC5, CPNE1, TTR* |
| CC | GO:0005913 | cell-cell adherens junction | 110 | 5 | 8.74E-05 | 0.0063 | *CXADR, ANXA2, CAMSAP3, ANXA1, MYH9* |
| **Note:** CC. GO-Cellular Component; GO. gene ontology; FDR. false discovery rate. | | | | | | | |
