# Supplementary material for: White matter deficits in cocaine use disorder: convergent evidence from in vivo diffusion tensor imaging and ex vivo proteomic analysis
Source: Transl Psychiatry. 2021 Apr 29;11:252. doi: 10.1038/s41398-021-01367-x (PMC8081729; doi:10.1038/s41398-021-01367-x)
Supplement: Supplementary file 4 — Supplementary Table 3 [file 41398_2021_1367_MOESM4_ESM.docx]

| **Supplementary Table 3.** Significantly differentially expressed proteins with adjusted *p*-value threshold | | | | | |
| --- | --- | --- | --- | --- | --- |
| **Symbol** | **ID** | ***p*-value** | **Adjusted *p*-value** | **LogFC** | **Alteration in CUD group** |
| SRSF3 | P84103 | 3.92E-05 | 4.71E-13 | 3.744589443 | Upregulation |
| CSNK2A2 | P19784 | 0.00121497 | 0.000421707 | -1.97645037 | Downregulation |
| MAP3K12 | Q12852 | 0.001925158 | 0.002449392 | 2.396067613 | Upregulation |
| TECPR1 | Q7Z6L1 | 0.002947587 | 0.010516611 | 1.328572275 | Upregulation |
| MT1E | P04732 | 0.004072912 | 0.021741065 | 1.861934227 | Upregulation |
| ARF5 | P84085 | 0.0041123 | 0.022084188 | -2.33179733 | Downregulation |
| LUM | P51884 | 0.004565729 | 0.025663338 | 1.729246043 | Upregulation |
| MMAB | Q96EY8 | 0.004602852 | 0.025925957 | -1.5607135 | Downregulation |
| MTMR2 | Q13614 | 0.004969282 | 0.029675545 | 1.400751579 | Upregulation |
| HSD17B12 | Q53GQ0 | 0.005360367 | 0.034099465 | -1.18027188 | Downregulation |
| IGHG4 | P01861 | 0.005844939 | 0.040700718 | 1.888207445 | Upregulation |
| **Note:** Between group comparisons, CUD vs Control; Adjusted p-value <0.05 and \|logFC\| > log2(1.5). | | | | | |
